# Frequency of Psychiatric Disorders in Suicide Attempters: A Cross-Sectional Study from Low-Income Country

**DOI:** 10.7759/cureus.14669

**Published:** 2021-04-24

**Authors:** FNU Pooja, Payal Chhabria, Pardeep Kumar, FNU Kalpana, Pardeep Kumar, Abbas Iqbal, Zoya Qamar, Dua Khalid, Amber Rizwan

**Affiliations:** 1 Internal Medicine, Chandka Medical College, Larkana, PAK; 2 Medicine, Liaquat University of Medical and Health Sciences, Jamshoro, PAK; 3 Internal Medicine, Shaheed Mohtarma Benazir Bhutto Medical University, Larkana, PAK; 4 Medicine, Jinnah Sindh Medical University, Karachi, PAK; 5 Internal Medicine, Ayub Teaching Hospital, Abbottabad, PAK; 6 Internal Medicine, Jinnah Sindh Medical University, Karachi, PAK; 7 Family Medicine, Jinnah Post Graduate Medical Center, Karachi, PAK

**Keywords:** suicide risk, psychiatry, suicide attempter

## Abstract

Introduction: In Pakistan, due to legal and religious association, cases of attempted suicides are underreported in Pakistan, yet it is essential to have accurate data so that the causality leading to this national tragedy can be studied and minimized. Psychiatric disorders leading to suicide is largely neglected and under-researched in Pakistan. In this study, we aim to observe the frequency of psychiatric disorders among suicide attempters, which can help the doctors to counsel and treat the patients better and devise preventive strategies.

Method: In this cross-sectional survey, patients brought to emergency with attempted suicide were enrolled in the study, after taking informed consent from the attendant. After initial treatment, the patient's clinical history was sought via a General Health Questionnaire-28. Once recognized, participants underwent detailed psychiatric evaluation and mental state examination.

Results: Three hundred and fifty-two (352) patients were brought to the emergency with attempted suicide, of which 249 (70.7%) patients were identified with psychiatric morbidity. The most common psychiatric disorders were mood disorder (32.1%), comorbid psychiatric disorder (20.4%), and anxiety disorders (18.4%). Our study also showed that the prevalence of comorbid psychiatric disorders was significantly higher in females as compared to males, whereas substance use disorder was more common in males.

Conclusion: The suicide rate has increased globally due to associated psychiatric disorders. Patients inflicting self-harm or failing at suicidal attempt are inclined towards attempting suicide in future. However, the social stigma associated with psychiatric disorders has heavily affected the process of successfully identifying and treating such patients. Along with focused long-term treatment, follow-up, and enhanced surveillance programs, mass awareness campaigns should be conducted to improve the knowledge and outlook of the general population towards psychiatric disorders.

## Introduction

According to a World Health Organization (WHO) report, approximately 800,000 people die of suicide annually; and for each suicide, as many as 20 more attempt suicide. The majority of them belong to low- or middle-income countries [1]. The suicide rate is alarmingly rising in Pakistan as well; as per the report of the Human Rights Commission in 2000, the average number of suicides increased to 250 per month from 175 per month in the previous year [2].

A previous suicide attempt is a high-risk factor for subsequent suicide and insight from unsuccessful attempters can be of immense value to prevent and curtail future suicides [3,4]. The demographic data of suicide in Pakistan show that it is most common in single men and married women, especially in people younger than 30 years of age. Domestic issues and relationship troubles are found to be the most common cause [5]. Psychiatric issues are receiving considerable attention globally as mental pain due to depression and hopelessness and comorbid psychiatric conditions including personality and mood disorders are a major contributing factor towards suicide attempt [6-8].

Due to legal and religious implications, the attempted suicides are underreported in Pakistan but it is imperative to collect accurate data so that causality leading to this national tragedy can be studied and minimized. The subject of psychiatric disorders leading to suicide is largely neglected and under-researched in Pakistan. In this study, we aim to observe the frequency of psychiatric disorders among suicide attempters, which can help the doctors to counsel and treat the patients better and devise preventive strategies.

## Materials and methods

This observational cross-sectional study was conducted in the emergency unit of a tertiary care hospital in Pakistan from January 2019 to March 2020. Three hundred and fifty-two (352) patients brought to the emergency with attempted suicide were enrolled in the study, after informed consent was sought from the attendant. The ethical review board of the institute approved patient enrolment. Patients were enrolled via consecutive convenient non-probability sampling.

After initial treatment, detailed history was taken to identify psychiatric morbidity. The questionnaire included General Health Questionnaire-28 (GHQ-28). Out of 352 people with attempted suicide, 249 (70.7%) participants were identified as having psychiatric morbidity after the initial assessment, which was included in the final analysis. They underwent detailed psychiatric evaluation and mental state examination using the criterion of Diagnostic and Statistical Manual of Mental Disorders, Fourth Edition Text Revised (DSM-IV).

Statistical analysis was done using SPSS v. 23.0 (IBM Corporation, Armonk, New York, USA). Data were presented as frequency and percentages.

## Results

The study included 249 suicide attempts identified with psychiatric morbidity; out of them, 151 (60.6%) males and 98 (39.1%) females were enrolled. The mean age of participants was 35 ± 09 years. The most common identified psychiatric disorders were mood disorder (32.1%), comorbid psychiatric disorder (20.4%), and anxiety disorders (18.4%). Comorbid psychiatric disorder was more common in females (28.5% vs. 14.5%, p-value: 0.007), while the substance use disorders were more common in males (16.5% vs. 3.06%; p-value; 0.0009) (Table 1).

**Table 1:** Gender-wise comparison of psychiatric disorders among suicide attempters ^a^Comorbid psychiatric disorders were defined as having more than one psychiatric disorder ^b^Organic mental disorder was defined as the decline in cognitive function due to disorders that are not psychiatric in nature

S. No	Psychiatric Diagnosis	Total cases (N=249) N (%)	Male (N=151) N (%)	Female (N=98) N (%)	p-value
1	Mood disorders	80 (32.1%)	51 (33.7%)	29 (29.5%)	0.47
2	Comorbid psychiatric disorder^a^	51 (20.4%)	22 (14.5%)	28 (28.5%)	0.007
3	Anxiety disorders	46 (18.4%)	25 (16.5%)	21 (21.4%)	0.33
4	Substance use disorders	28 (11.2%)	25 (16.5%)	03 (3.06%)	0.0009
5	Personality disorders	19 (7.6%)	08 (5.2%)	11 (11.2%)	0.08
6	Schizophrenia	16 (6.4%)	12 (7.9%)	04 (4.08%)	0.22
7	Organic mental disorders^b^	05 (2.0%)	04 (2.6%)	01 (1.02%)	0.37
8	Other psychotic disorders	05 (2.0%)	04 (2.6%)	01 (1.02%)	0.37

## Discussion

The most common psychiatric disorder in our study was mood disorder (32.1%), followed by comorbid psychiatric disorder (20.4%) and anxiety disorder (18.4%). Others such as schizophrenia, substance use, and personality disorders were also observed in some of the suicide attempters. The prevalence of comorbid psychiatric disorders was found to be significantly higher in females, whereas substance use disorder was more common in males.

Prior studies on similar topics have proved that mood disorder is strongly related to suicide and suicidal behavior [9]. Chances of committing suicide throughout life in people with mood disorders are approximately 10 times higher than in people with no underlying psychiatric conditions [10]. There is a debate on these values being quite higher than the actual ones [11], but people with mood disorders are more likely to self-harm [10], with 25%-50% of them trying to attempt suicide [12]. There is evidence that self-harm is known to trigger people to attempt suicide; therefore, it plays a major role in suicidal behavior [13].

In the early 21st century, depression was found to be the most common cause of unnatural death all around the world, followed by substance use disorders, schizophrenia, and personality disorders [14]. However, there were variations between in- and outpatients. Forty-five percent of patients who committed suicides during their stay in the hospital were reported to have schizophrenia and organic mental disorders; 32% of the outpatient suicides had depression, substance use disorders, anxiety, and adjustment disorders. In terms of depression, both groups were on the same page [15]. This indicates that people with suicidal behavior or those who commit suicide are shown to have similar problems as mentioned in our study.

Suicide and mental health disorders are labeled as taboo in our society. Due to this, people do not step forward to seek help and avail the services and treatment needed to prevent these problems [16-18]. Keeping in mind the increasing number of suicides, it is extremely significant to seek a solution to this problem. Research has shown that although public awareness campaigns are the primary approach to talk about sensitive topics and to spread knowledge on the importance of seeking professional health, it has not shown definite results [19,20]. On the contrary, mass media campaigns are known to be the most effective medium in rectifying suicidal behavior and spreading awareness among the people who are more likely to attempt suicide [20]. Moreover, people who have attempted suicide should seek a therapist with consistency. Doctors should keep a check on the patients who are more likely to commit suicide again and should foremost provide emotional help in addition to the medications.

To the best of our knowledge this is the first study in a local setting to study the prevalence of psychiatric disorders in suicide attempters. However, the study has its limitation as well. Since it was a cross-sectional study, a definite association could not be established. Secondly, since all participants were taken from a single institute, sample size diversity was reduced.

## Conclusions

Mood disorders, anxiety, and comorbid psychiatric disorder were commonly presented in suicide attempters in our study. With developing times and modern problems, the suicide rate has increased globally due to associated psychiatric disorders. However, the social stigma associated with psychiatric disorders has heavily affected the process of successfully identifying and treating such patients. Patients inflicting self-harm or failing at suicidal attempt are inclined towards attempting suicide in future. Therefore, there is a dire need for focused long-term treatment, follow-up, and enhanced surveillance programs in order to make mental health care services accessible to everyone. Mass awareness campaigns should be conducted to improve the knowledge and outlook of the general population towards psychiatric disorders and the population in distress should be actively encouraged to seek psychiatric help for better long-term outcomes.
